# Point-of-care potentials of lateral flow-based field screening for *Mycoplasma bovis* infections: a literature review

**DOI:** 10.1093/biomethods/bpae034

**Published:** 2024-05-22

**Authors:** Ilemobayo V Fasogbon, Erick N Ondari, Tusubira Deusdedit, Loganathan Rangasamy, Sasirekha Krishnan, Patrick M Aja

**Affiliations:** Department of Biochemistry, Kampala International University-Western Campus, Bushenyi 41201, Uganda; Centre for Biomaterials, Cellular and Molecular Theranostics (CBCMT), Vellore Institute of Technology, Vellore 632014, India; Department of Biological Sciences, School of Pure & Applied Sciences, Kisii University, Kisii 40200, Kenya; Department of Biochemistry, Mbarara University of Science and Technology, Mbarara 40301, Uganda; Centre for Biomaterials, Cellular and Molecular Theranostics (CBCMT), Vellore Institute of Technology, Vellore 632014, India; Centre for Biomaterials, Cellular and Molecular Theranostics (CBCMT), Vellore Institute of Technology, Vellore 632014, India; Department of Biochemistry, Kampala International University-Western Campus, Bushenyi 41201, Uganda

**Keywords:** *Mycoplasma bovis*, assay, lateral flow, diagnosis, systematic review

## Abstract

Point-of-care (POC) field screening for tools for *Mycoplasma bovis* (*M. bovis)* is still lacking due to the requirement for a simple, robust field-applicable test that does not entail specialized laboratory equipment. In accordance with the Preferred Reporting Items for Systematic Reviews and Meta-analysis (PRISMA) guidelines, this review identifies the methodologies that were retrieved based on our search strategy that have been reported for the diagnosis of *m. bovis* infection between 2014 and diagnostics. A search criterion was generated to curate 103 articles, which were reduced in number (to 46), following the screening guidelines of PRISMA. The 43 articles included in the study present 25 different assay methods. The assay methods were grouped as microbiological culture, serological assay, PCR-based assay, LAMP-based assay, NGS-based assay, or lateral flow assay. We, however, focus our discussion on the three lateral flow-based assays relative to others, highlighting the advantages they present above the other techniques and their potential applicability as a POC diagnostic test for *M. bovis* infections. We therefore call for further research on developing a lateral flow-based screening tool that could revolutionize the diagnosis of *M. bovis* infection.

## Introduction

*Mycoplasma bovis* is one of the foremost causes of bovine respiratory disease (BRD), a major health problem which affects both adult and calf cattle, and has a pronounced economic impact on the cattle industry [1]. BRD causes economic losses in herds due to reduced productivity and increased costs of treatment and culling. *Mycoplasma bovis* was initially isolated in 1961 from a severe case of mastitis in a dairy herd experiencing an outbreak in the USA, but it is now known to cause various clinical symptoms in cattle, including mastitis, pneumonia, and arthritis. *Mycoplasma bovis* is also linked to the global etiology of bovine mycoplasmosis. It is a pathogen of economic importance to the cattle industry (Fu, Sun, Zhang, et al., 2014). Similar to other members of the Mycoplasma genus, *M. bovis* lacks a cell wall, with a genome size of around 953,114 bp and less than 30% GC content [3]. *Mycoplasma bovis* is presently acknowledged as one of the primary and commonly isolated Mycoplasma species associated with cattle disease globally [4].

*Mycoplasma bovis* infections lack effective treatment, whereas moderate infections in cattle have the potential to cause an infection with severe clinical manifestations, as well as difficulty in diagnosis [3, 4]. *Mycoplasma bovis* infections spread rapidly in cattle herds, making *M. bovis* more important. One cattle infected with the pathogen could be a source of the infection within a herd and also transmit the infection, especially to closely grazing herds for years; therefore, identifying, isolating, and culling infected cattle is the pragmatic step to curb the spread of *M. bovis* infections [5]. Rapid, sensitive, and accurate screening of the herd is needed to control potential outbreaks. Previous review on *M. bovis* diagnostic identified microbial culture, serology, DNA-based, and mass spectrometry as the broad category of diagnostic techniques currently available for *M. bovis* infection, outlining the merits and demerits of each one [4, 6]. In this study, we methodically studied the potential of lateral flow-based diagnostic techniques for POC rapid screening for *M. bovis*, with a call for further research to aid its field usability.

## Materials and methods

A comprehensive literature search of published articles on the diagnosis and detection of *M. bovis* was carried out on WoS and Scopus databases on 18 June 2023. The following search terms were used: “Mycoplasma bovis,” “detection,” “diagnosis,” “diagnostics” and “assay,” “testing,” and “screening”. Delimiters like Boolean operators (AND/OR), quotation marks, parentheses, wildcards, and asterisks (*) were used to combine the search terms as (diagnos* OR detect* OR test* OR assay* OR screen*) AND (“mycoplasma bovis”). The search field was limited to “Title” in both databases because of the specificity of the review focus and the robustness of the search terms. Records that meet the inclusion/exclusion criteria (Table 1) were downloaded for screening. A systematic paper selection process comprising title, abstract, and full-text screening was sequentially carried out in accordance with the Preferred Reporting Items for Systematic Reviews and Meta-analyses (PRISMA) 2020 guideline [7–9].

**Table 1.** Inclusion and exclusion criteria.

Parameter	Inclusion criteria	Exclusion criteria
**Study design**	Only original articles will be included	Other publications aside from original articles, including case reports, letters to the editor, conference abstracts, opinion articles, and review articles, will be excluded
**Diagnosis**	Studies focusing on the diagnosis of *M. bovis* infection using any diagnostic method, including laboratory tests, will be included	Studies that only describe imaging techniques and clinical signs/symptoms for diagnosing *M. bovis* infection will be excluded.
**Detection**	Studies that report or discuss detection methods for *M. bovis* or any biomarkers of *M. bovis* infection will be included	Studies that do not report or discuss detection methods for *M. bovis* or any biomarkers of *M. bovis* infection will be excluded.
**Language**	Studies published in English will be included	Studies published in languages other than English will be excluded
**Publication date**	Only studies published in 2014-2023 will be included	Studies published in 2014 and after 18^th^ June 2023, when the search was carried out, will be excluded

## Results

### Search results

**Figure 1.**
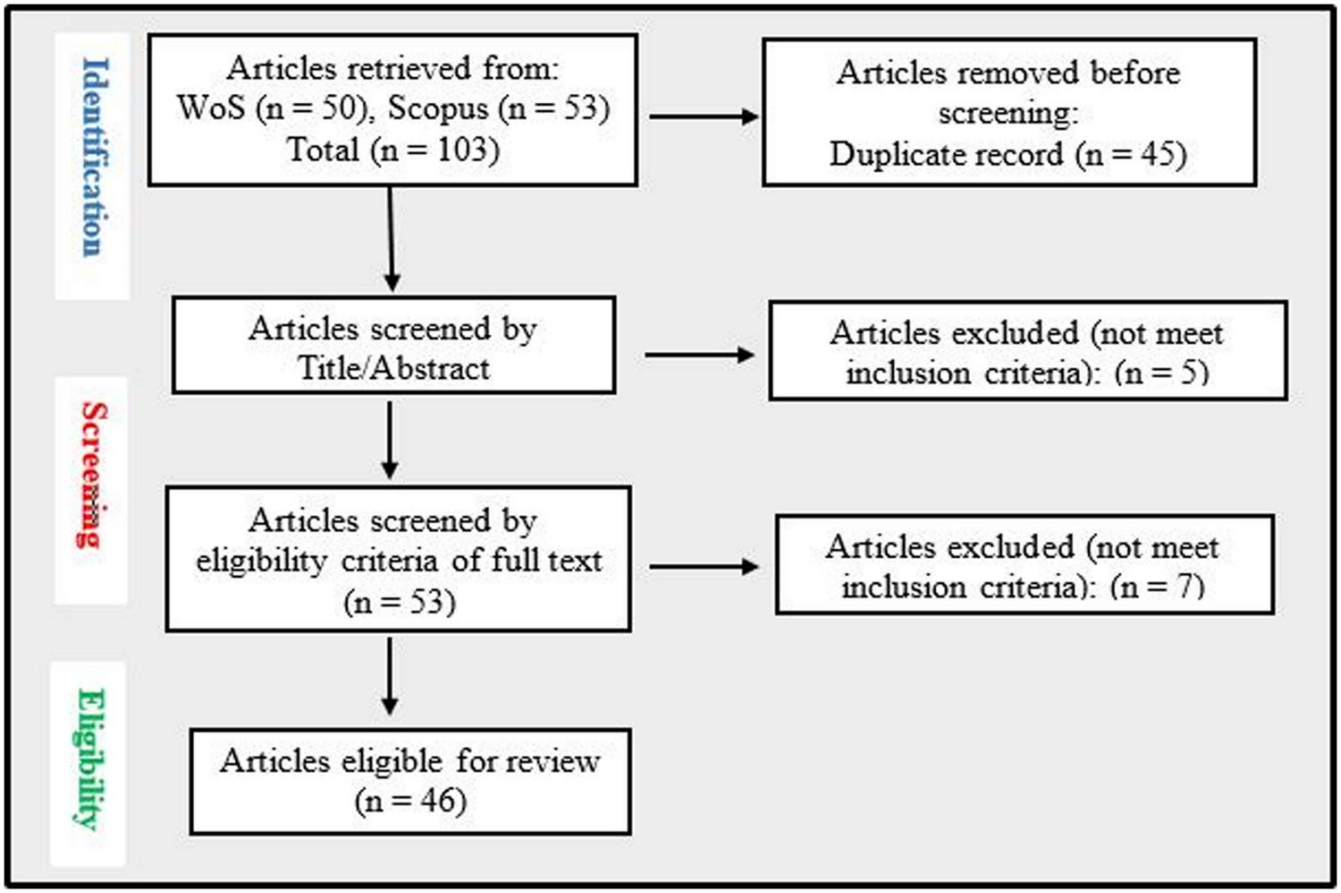
PRISMA study selection flow chart.

WoS search recognized 50 articles, and Scopus search returned 53 articles, totaling 103 articles from both databases. The results from each database were exported in BibTeX format. Then, merged and tidied to remove duplicated articles on: https://flamingtempura.github.io/bibtex-tidy/. Forty-three duplicated articles were removed. The 58 records left were initially screened by their titles and abstracts (five articles screened out). Then, a more rigorous full-text screening identified seven other articles that did not meet the inclusion criteria. A total of 12 articles were excluded in accordance with the eligibility criteria, and 46 articles were reviewed in the study, having passed the eligibility criteria and quality assessment (Fig. 1).

### Summary of detection/diagnostic techniques for *M. bovis* infection

Articles that reported methods used in the detection or/and diagnosis of *M. bovis* between 2014 and 2023 were reviewed in this study (Table 2). The methods can be grouped as microbiological culture (six articles), serological assays (27 articles), PCR and PCR-related assays (19 articles), LAMP and LAMP-based assays (six articles), NGS-based diagnostics (two articles), lateral flow assays (three articles). Some of the articles utilized and compared two or more methods, especially comparing the sensitivity and specificity of the traditional microbiological culture with other methods [10–15] or compare different commercially available ELISA kits [16–19]. Such articles are, therefore, cited under all such categories. This may be a total of 63 method-based categorizations of 46 articles.

**Table 2.** All Assay methods used to diagnose *M. bovis* infection between 2014 and 2023

Methods	Specific assay	Study Reference	No
**Microbiological culture**		(Hazelton et al., 2018, 2020; Jaramillo et al., 2023; Parker et al., 2017; Salina et al., 2020; Szacawa et al., 2016)^10,11,12,13,14,15^	6
**Serological assay**	BIO K302 ELISA and BIO K260 Commercial ELISA Kit	(Akan et al., 2014; Andersson et al., 2019; Nielsen et al., 2015; Parker et al., 2017; Petersen et al., 2018, 2020; Salgadu, Cheung, et al., 2022; Schibrowski et al., 2018; Veldhuis et al., 2023; Vojinović et al., 2014)^11,16,17,18,19,20,21,22,23,24^	
			10
	ID Screen Commercial ELISA Kit	(Andersson et al., 2019; Petersen et al., 2018, 2020; Veldhuis et al., 2023)^16,17,18,23^	4
	MilA-based ELISA	(Al-Farha et al., 2020; Petersen et al., 2018; Salgadu, Cheung, et al., 2022; Salgadu, Firestone, et al., 2022; Wawegama et al., 2014, 2016)^19,20,25,26,27,28^	6
	Optimized iELISA	(Pires et al., 2021)^29^	1
	rMbovP579-based ELISA	(Khan et al., 2016)^30^	1
	IgG avidity test	(Han et al., 2015)^31^	1
	Western Blotting	(Schibrowski et al., 2018)^22^	1
	Dc-ELISA	(Fu et al., 2014a)^2^	1
	AgELISA	(El-Tawab et al., 2019)^33^	1
	Dc-ELAA	(Fu, Sun, Yu, et al., 2014)^32^	1
	**Total count for serological assays**		**27**
**PCR and PCR-related Assays**	Conventional PCR	(Akan et al., 2014; Andersson et al., 2019; Cengiz et al., 2021; Hamad et al., 2019; Hazelton et al., 2020; Jaramillo et al., 2023; Junqueira et al., 2020; Parker et al., 2017; Salina et al., 2020; Szacawa et al., 2016)^10,11,13,14,15,18,24,34,35,36^	10
	Real-time	(Behera et al., 2018; Buckle et al., 2020; Jaramillo et al., 2023; Nielsen et al., 2015; Surýnek et al., 2016; Wisselink et al., 2019)^15,23,37,39,40^	6
	multiplex qPCR	(Chauhan et al., 2021)^41^	1
	Taqman real-time PCR	(Naikare et al., 2015)^42^	1
	PCR/DGGE	(Szacawa et al., 2016)^10^	1
	**Total count for PCR and PCR-related assays**		**19**
**LAMP and LAMP-based assays**	LAMP	(Appelt et al., 2019)^43^	1
	real-time LAMP	(Ashraf et al., 2018; Fan et al., 2018; Pan et al., 2020)^44,45,46^	3
	PURE-LAMP	(Itoh et al., 2020)^47^	1
	Improved LAMP	(Higa et al., 2016)^1^	1
	**Total count for LAMP and LAMP-based assays**		**6**
**NGS-based diagnostics**	Nanopore	(Bokma et al., 2021)^48^	1
	i-Seq (Illumina)	(Liapi et al., 2021)^49^	1
	**Total count for NGS-based Diagnostics**		**2**
**Lateral flow assay**	CNPs-based LFS	(Shi et al., 2020a)^50^	1
	RPA-LFD and LFS RPA	(Zhao et al., 2018; Li et al., 2021)^51,52^	2
	**Total count for lateral flow assays**		**3**

MilA—mycoplasma immunogenic lipase A; Ag-ELISA—antigen-detection enzyme-linked immunosorbent assay; Dc-ELISA—direct competitive enzyme-linked immunosorbent assay; ELAA-enzyme-linked aptamer assay; NGS—next generation sequencing; PURE—purified enzymes; LAMP—loop-mediated isothermal amplification; PCR—polymerase chain reaction; DGGE—denaturing gradient gel electrophoresis; CNP—carbon nanoparticle; LFS—lateral flow strip; LFD—lateral flow dipstick; RPA—recombinase polymerase amplification.

### Summary of other assay methods and their major limitations

**Figure 2.**
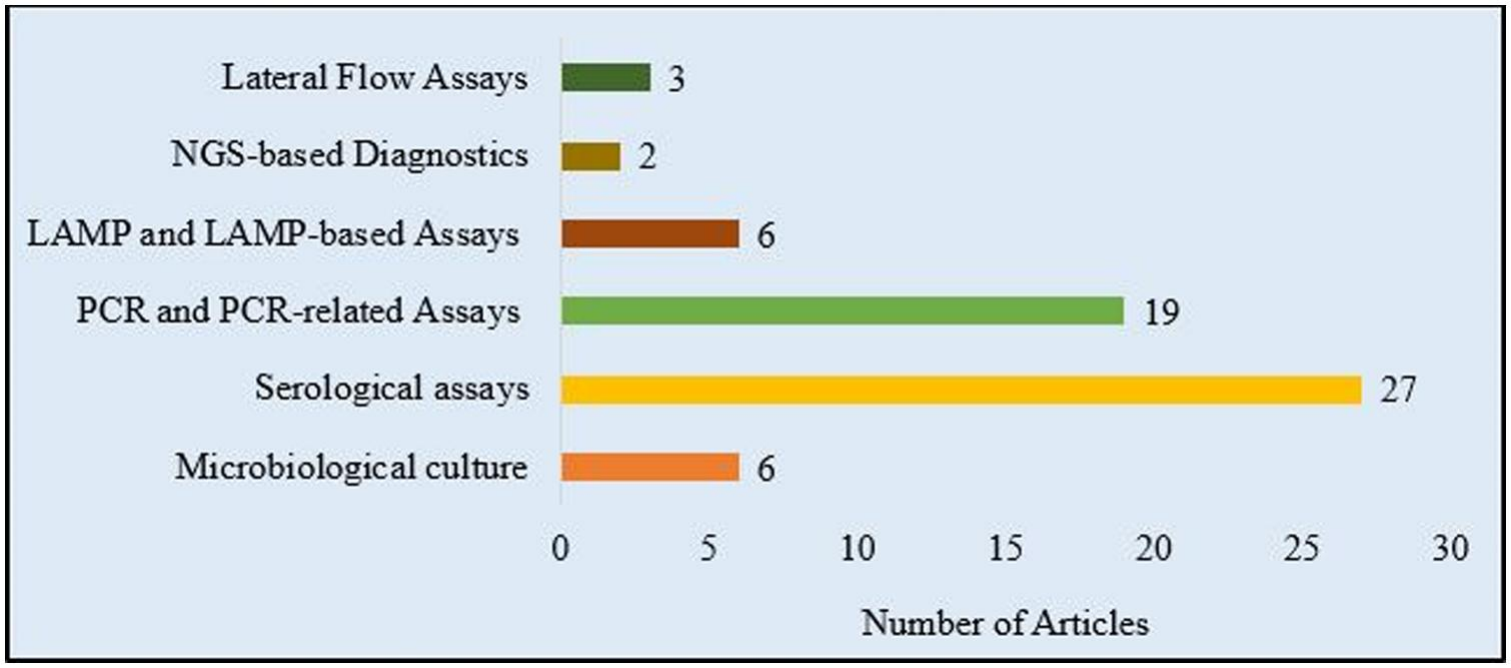
Categories of diagnostic assays reported for *M. bovis*

Generally, diagnostic assays are used to determine the presence or absence of a particular disease or condition in an individual [38]. The 46 articles reviewed in the research employed various assay methods, categorized in Fig. 2. Having a focus on the three lateral flow assay techniques identified in the search, we first highlighted the basic principles and limitations of the other reported methods. Thereafter, the lateral flow techniques are described, as well as their potential and possible applications and improvements.

#### Microbiological culture

The microbiological culture method for diagnosing *M. bovis* infection involves the isolation and propagation of the pathogen from clinical specimens, such as nasal swabs, lung tissues, or milk samples. *Mycoplasma bovis*, being a fastidious bacterium lacking a cell wall, requires specialized culture conditions for successful isolation. *Mycoplasma bovis* colonies are typically small, pinpoint-sized, and appear as tiny, dome-shaped structures. These colonies can be observed under a microscope or through visual inspection. This approach provides a valuable tool for understanding the prevalence and epidemiology of *M. bovis* in livestock populations, aiding in the implementation of targeted control and prevention strategies.

Microbiological culture can be time-consuming, typically taking several days to obtain results. It is not every infected animal in dairy herds that exhibits symptoms of the disease. It is, therefore, challenging to identify carriers or sub-clinically infected animals since there isn't a constant location of infection to sample. Subclinical mastitis can be difficult to diagnose since *M. bovis* shedding in milk occurs sporadically. There is also difficulty in identifying subclinical infection in non-lactating stock [12]. More importantly, the simplicity of *M. bovis*, like other mycoplasmas, makes them impairs their ability to synthesize amino acids and fatty acids, hence their fastidious nutritional requirements [4]. Isolating *M. bovis* by culture is therefore often compromised by the overgrowth of other faster growing bacteria [10]. Also, the organisms may lose viability during sample collection, transportation, or storage, especially if not handled under optimal conditions. This can result in false-negative culture results. Culture may not always differentiate between multiple microbial species present in a clinical sample [11]. This can complicate the interpretation of results, especially if one pathogen inhibits the growth of another. Microbiological culture requires skilled laboratory personnel and involves a series of labour-intensive steps, from sample inoculation to result interpretation. This can increase the likelihood of errors and the overall cost of testing.

#### Serological assay

The serological assay for diagnosing *M. bovis* infection relies on the detection of specific antibodies produced by the host in response to the pathogen. This method provides a rapid and efficient means of identifying the exposure and infection status of herds, both to delimit and to confirm the absence of *M. bovis* [53], which provide valuable information for both diagnostic and surveillance purposes.

Serological assays play a crucial role in diagnosing *M. bovis* infections because they specific antibodies produced by the host. These assays are valuable for surveillance, monitoring the spread of the pathogen, and assessing the effectiveness of control measures in animal populations. Serological assays are reliant on the host's immune response, which may take time to produce detectable levels of antibodies [22]. Moreover, antibodies can persist for an extended period of time after an infection has been resolved. Detecting antibodies does not necessarily indicate an active infection, and the presence of antibodies may represent a past exposure or a successfully cleared infection. Cross-reactivity can occur when antibodies recognize antigens from closely related microorganisms, like *M. agalactiae* [54]. This may lead to false-positive results or difficulty in distinguishing between different pathogens [55, 56]. Furthermore, host factors such as age, immune status, and genetic variability can impact the antibody response. Some individuals may produce antibodies more rapidly or at higher levels than others, influencing the assay results.

#### Molecular technique

The molecular technique for diagnosing *M. bovis* infection employs nucleic acid amplification methods, such as polymerase chain reaction (PCR) and loop-mediated isothermal amplification (LAMP), to detect and amplify specific genetic material unique to the pathogen. High-throughput sequencing sometimes follows the amplification to identify and analyze the pathogen's nucleotide sequence. This method has high sensitivity and specificity, allowing for the rapid and accurate identification of *M. bovis* in clinical samples. The approaches can exclude cross-reactivity with other related bacteria and Mycoplasma species [39]. El-Tawab et al. [33], however, recommended that culturing milk samples before PCR improved the sensitivity. Molecular techniques are instrumental in early detection, accurate diagnosis, and monitoring of *M. bovis* prevalence in animal populations. Itoh et al. [47] evaluated LAMP as a more rapid, simple, and accurate detection method to directly detect the *M. bovis* gene in milk.

Molecular techniques are rigorous, and contamination during sample collection, handling, or laboratory use can lead to false-positive results. Molecular techniques detect genetic material that may persist even in non-viable microorganisms. This could result in false-positive results. The possibility of genetic diversity within microbial species can affect the ability of primers to bind and amplify target sequences. Molecular diagnostic methods can be costly to implement. This may limit their accessibility in resource-limited settings. Performing molecular diagnostics requires specialized training, both in sample processing and in data analysis. Skilled personnel are essential to ensure the reliability and accuracy of results.

A molecular technique used in mass spectrometry, matrix-assisted laser desorption/ionization time-of-flight (MALDI-TOF) has proved reliable and accurate as it identifies *M. bovis* by analyzing their protein profiles, ionizing and measuring their mass-to-charge ratio [57]. McDaniel & Derscheid [58] combined MALDI-TOF mass spectrometry with high-resolution melting PCR to detect genetic variations by monitoring the DNA strands melting after amplification. The outcome holds great promise for a swift and regular diagnosis of *M. bovis*. However, like other molecular techniques, cost, dependence on skilled personal and specialized equipment limit its potential as in POC diagnostics.

#### NGS-based assay

NGS is a high-throughput DNA sequencing technology that allows the simultaneous sequencing of millions of DNA fragments. NGS has revolutionized genomics research and clinical diagnostics, providing unprecedented insights into genomic information. The principle involves the parallel sequencing of short DNA fragments, which are then computationally reconstructed to reveal the complete sequence of the target DNA [48, 49]. Sample contamination, high cost, the need for sophisticated bioinformatics tools, skilled analysts, and error rates are the major limitations of this technique.

### Lateral flow assay techniques for *M. bovis* diagnosis

In general, LFAs offer significant advantages in diagnostics, making them particularly suitable for POC applications. Such advantages include: the fact that they are often designed for ease of use in various settings, including remote or resource-limited areas [9, 59]. They provide rapid results at the POC, facilitating quick decision-making without the need to send samples to a centralized laboratory. Also, LFAs are generally cost-effective compared to more complex laboratory-based methods. They are typically user-friendly and require minimal specialized training. Healthcare professionals, as well as individuals without extensive laboratory expertise, can perform these assays with relative ease. LFA testing tools are often compact and portable in nature to enhance their applicability in diverse settings, as they can be easily transported and deployed in areas with limited infrastructure, enabling on-the-spot testing without the constraints of a fixed laboratory environment. Various diagnostic purposes, such as the detection of antibodies, antigens, and nucleic acids, can adapt LFAs for their versatility. This versatility makes them valuable tools for a wide range of infectious diseases, pregnancy testing, and other health-related assessments [60].

Two of the three lateral flow-based assays that were identified from the search strategies employed recombinase polymerase amplification (RPA) to amplify the DNA of *M. bovis* to enhance its subsequent detection by a probe-based lateral flow strip. The third lateral flow-based assay was developed to detect antibodies at *M. bovis* in the host.

#### The RPA-FLS

The RPA technique has become a promising isothermal DNA amplification rapid assay that could be useful in resource-limited settings. Zhao et al. [51] introduced an assay technique that combines RPA and lateral flow dipstick (LFD) for *M. bovis* detection*.* The combined technique provides rapid and easy detection of *M. bovis* DNA. With a detection limit of 20 copies per reaction, the assay successfully detected *M. bovis* DNA in 30 min at 39°C, which was comparable with the quantitative PCR (qPCR) assay.

The working principle of the RPA-FLD involves amplification (RPA), detection (LFD), and visualization [61, 62]. The uvrC and oppD-oppF genes were amplified from the genomic DNA extracted from *M. bovis* reference type strain PG45 using specialized forward and reverse primer pairs. As the recombinase enzyme unzips the DNA, polymerase makes new copies of the target genes. The amplified DNA is then applied to the LFD, which is a special strip. This strip was constructed with an immobilized probe, a molecule that can capture the amplified *M. bovis* gene. If the *M. bovis* DNA is present, the corresponding genes would be amplified by the RPA and captured and detected by the LFD. The dipstick features markers that undergo a color change upon capturing the target DNA. This color change is visible to the naked eye, providing a quick and easy way to confirm the presence of *M. bovis* DNA. The RPA-LFD showed 99.00% sensitivity, 95.61% specificity, and 0.902 kappa coefficient compared with the qPCR.

The study by Li et al. [52] looked at the uvrC gene of *M. bovis* and compared a real-time RPA assay (monitored by fluorescence) and an RPA with a lateral flow strip (LFS) assay. The real-time RPA in a Genie III took 20 minutes to complete at 39°C, whereas the LFS-RPA in an incubator block took 15 minutes. The lateral flow strip displayed the results within 5 minutes. High specificity for *M. bovis* was seen in both assays, and there was no cross-reaction with the other examined pathogens. The authors concluded that, as an intriguing and promising instrument, the developed RPA assays could efficiently, conveniently, and credibly detect *M. bovis* in bovine milk, and the assays would be beneficial in the quick response to *M. bovis* infection, causing bovine mastitis.

Generally, people consider RPA in conjunction with LFS to be a relatively simple and portable method, using the LF probe to avoid the challenges of multiplexing and non-specific amplification that RPA frequently faces. Yet, the technique still requires basic laboratory equipment, such as a heat block or water bath, for the amplification step [61]. The RPA reaction requires incubation at temperatures between 35°C and 42°C for 15-30 minutes [51, 52]. Although designed for simplicity, effective implementation of RPA-LFD may still require some level of user training. The cost of reagents and consumables may also have an impact on the feasibility of widespread POC usage.

#### The CNP-LFS

Whereas RPA-LFD incorporates a lateral flow strip for detecting *M. bovis* DNA, the CNP-LFS of Shi et al. [50] was developed to detect antibodies against *M. bovis* sequel to infections. Carbon nanoparticles (CNPs) were used as the labeled materials, as in previous studies [63, 64]. The intense black color of CNPs provides good contrast for visual detection. The results from the test strip were highly consistent with those from ELISA [50]. The test showed high specificity (100%) and no cross-reaction with other bovine pathogens. The detection sensitivity of the test was also relatively high (97.67%). According to the authors, all the results indicated that the colloidal carbon test strip could serve as a simple, rapid, sensitive, and specific diagnostic method for detecting antibodies against *M. bovis* at cattle farms.

However, developing a lateral flow detection assay to detect antibodies against a pathogen, rather than detecting an antigen, biomarker, or pathogen DNA, comes with its own set of disadvantages. The immune system typically produces antibodies in response to infection, and their presence may lag behind the appearance of the pathogen or its antigens [65]. Detecting antibodies might result in a time lag between the onset of infection and a positive test result. Also, in the early stages of infection, the concentration of antibodies may be low or undetectable. This can lead to false-negative results, especially during the initial phase of an infection. Moreover, the diagnostic window for antibody detection may be narrower compared to the direct detection of antigens or DNA [30, 66]. Furthermore, antibodies persist for a longer duration, potentially leading to false positives or difficulty in differentiating past and active infections. The effectiveness of antibody detection also relies on the host's immune response, which can be influenced by factors such as immune suppression or variability in individual immune systems [67, 68].

## Future perspectives

Given the enormous advantages of the lateral flow-related assay, especially its potential as a POC rapid diagnostic tool for *M. bovis* infection, we suggest that further research in this direction could soon yield a more substantial result, revolutionizing the diagnosis of *M. bovis* infection. This discovery has the potential to enhance disease surveillance, enable timely intervention, and ultimately mitigate the economic and health impacts of *M. bovis*-related diseases in livestock, all while contributing to the overall health and sustainability of livestock operations. The development of POC rapid diagnostic tools for *M. bovis* infection represents a significant breakthrough that would pragmatically contribute toward the realization of the United Nations’ Sustainable Development Goals (UN-SDG) 2 and 3. UN-SDG 2 addresses Zero Hunger (goal 2); and Cattle, the host of *M. bovis* infection, is a source of food to many worldwide, whereas UN-SDG 3 addresses health for all. In accordance with the global one-health paradigm, it is essential to tackle *M. bovis* infection as the pathogen has been suggested to have zoonotic potential.

## Data Availability

The datasets/information used for this study are available from the corresponding author upon reasonable request.
